# Editorial for the Special Issue: “Honey Bee Products as an Alternative or Complement to Classical Antibiotics”

**DOI:** 10.3390/antibiotics10030234

**Published:** 2021-02-26

**Authors:** Piotr Szweda

**Affiliations:** Department of Pharmaceutical Technology and Biochemistry, Faculty of Chemistry, Gdańsk University of Technology, ul. G. Narutowicza 11/12, 80-233 Gdańsk, Poland; piotr.szweda@pg.edu.pl

Based on World Health Organization reports, the resistance of bacteria to well-known antibiotics is becoming a major global health challenge [1]. The number of bacteria and pathogenic fungi resistant to a plethora of antibiotics and chemotherapeutics has quickly increased during the last two to three decades, and very few new antimicrobial medicines have been approved since the beginning of the 21st century. Thus, there is an urgent need to search for new agents, as well as therapy systems effective in the treatment of infectious diseases. A promising but underestimated group of potential antimicrobial agents are bee products. 

This Special Issue includes four reviews and nine research articles focused mainly on the antimicrobial potential of bee products and possibilities of their application for the treatment of infectious diseases caused by different groups of bacteria. The articles have been prepared by around seventy authors from thirteen different countries, namely, Brazil, the Czech Republic, France, Greece, Italy, Malaysia, the Netherlands, Poland, Portugal, Romania, Spain, the UK and the USA. 

In review articles, Nolan and coworkers [2] and Combarros-Fuertes and colleagues [3] present the current state of the knowledge about the perspectives, advantages and limitations of the application of honey for the treatment of infections caused by antibiotic-resistant bacteria. Honeybee products are a great source of polyphenols, with a number of recognized bioactivities, such as antibacterial, antiviral, antiparasitic, anticancer, anti-inflammatory and antioxidant properties. Curutiu and coworkers [4] discussed the potential of polyphenols of honeybee origin in prophylactic and therapeutic approaches in oral pathologies, including bacterial and fungal infections. An excellent review on the antimicrobial activity and therapeutic potential of lesser-known bee products, namely, bee pollen and bee bread, is presented by Didaras and colleagues [5]. 

Still, very little is known about the role of phytochemicals in the antimicrobial activity of honey. Some important information in this area is presented in the article of Shirlaw and coworkers [6], who calculated the predicted binding affinities and ligand efficiencies of Manuka and Heather honey constituents for PaDsbA1, the main enzyme controlling the correct folding of virulence proteins in *Pseudomonas aeruginosa*. The group of Ng [7] revealed that honeydew honeys produced by stingless bees (*Heterotrigona itama* and *Geniotrigona thoracica*) exhibit higher antibacterial activity than the blossom and honeydew honeys produced by the honey bee (*Apis cerana*). Moreover, the authors observed some synergistic interactions between honey and selected antibiotics (ampicillin and gentamicin). The outcomes of the study of Yu and coworkers [8] reveal a high bactericidal activity and spore-inhibition effect of manuka honey against *Clostridioides difficile*, a major cause of antibiotic-associated diarrhea worldwide. Nairi et al. [9] discussed the beneficial effects of MGH (medical-grade honey) on infected diabetic ulcers. The authors present six patients with infected diabetic ulcers, some of whom were at risk of (further) amputation. Previous treatments with antibiotics, silver and alginate dressings, surgical closure, and maggot therapy were ineffective. MGH therapy reduced the malodor in a couple of days and controlled infection within 2–3 weeks; moreover, using MGH enhanced the process of wound healing.

The following two research articles concentrated on the antibacterial potential of bee propolis. Lavigne and colleagues revealed a synergistic effect of propolis and antibiotics [10] against uropathogenic *Eschericha coli*, and de Figueiredo and coworkers [11] found that extracts of Brazilian red propolis exhibit activity comparable with amoxicillin’s in controlling multispecies subgingival biofilms.

The outcomes of an investigation performed by Polish researchers [12] reveal the high antistaphylococcal activity of ethanolic extracts of bee pollen and bee bread. The authors also found that bee bread exhibits slightly higher potential compared to bee pollen, which is of particular importance from the point of view of the health of bee colonies. 

The two articles by Iorizzo and coworkers [13,14] concern slightly different—and very interesting—areas of the health benefit potential of bee products. The authors investigated the antimicrobial potential of lactic acid bacteria (LAB) isolated from the bee products and gastrointestinal tracts of *Apis mellifera* L. The investigated isolates exhibited promising activity against dangerous bee pathogens: *Ascosphaera apis* and *Paenibacillus larvae* (the causative agent of American foulbrood (AFB)). It is highly possible that LAB, isolated from bee products and also bees’ guts, produce metabolites that are effective inhibitors of the etiological agents of different infections of other animals and humans. 

The articles presented in this Special Issue are important for the promotion of the application of bee products as antimicrobial agents in clinical scenarios, but also to fill gaps in our knowledge about the molecular mechanisms of their biological activities. Some articles reveal that the microbiomes of bee products also constitute promising—but not yet well investigated—sources of microorganisms, producing metabolites of high antibacterial activity. Aspirant authors interested in the antimicrobial potential of bee products are invited to contribute to the second edition of the Special Issue with the title “Honey Bee Products as an Alternative or Complement to Classical Antibiotics”.
